# Analysis of microbial communities in solid and liquid pig manure during the fertilization process

**DOI:** 10.1038/s41598-023-50649-5

**Published:** 2024-01-02

**Authors:** Soo-Ryang Kim, Junkyung Lee, Myung Gyu Lee, Ha Guyn Sung, Sun-Goo Hwang

**Affiliations:** 1 Industry-Academic Cooperation Foundation, Sangji University, Wonju, 26339 Republic of Korea; 2 Department of Applied Plant Science, Sangji University, Wonju-si, 26339 Republic of Korea; 3 Department of Smart Life Science, Sangji University, Wonju-si, 26339 Republic of Korea; 4 Animal Feeding and Environment Laboratory, Department of Animal Science, Sangji University, Wonju-si, 26339 Republic of Korea

**Keywords:** Microbiology, Environmental sciences

## Abstract

Utilizing livestock manure as organic fertilizer in sustainable agriculture is crucial and should be developed through an appropriate manufacturing process. Solid–liquid separation contributes to reducing odor, managing nutrients in livestock excretions, and lowering the cost of transporting manure to arable soil. To investigate the impact of fermentation after solid–liquid separation, we examined the specific correlation between chemical properties and bacterial communities in solid–liquid manures before and after the fermentation process. In terms of chemical properties before fermentation, the levels of electrical conductivity, nitrogen, ammonium nitrogen (NH_4_^+^-N), potassium, sodium, and chloride were higher in the liquid sample than in the solid sample. However, the chemical components of the liquid sample decreased during fermentation, which could be attributed to the low organic matter content. Many chemical components increased in the solid samples during fermentation. Fifty-six bacterial species were significantly correlated with NH_4_^+^-N and phosphorus. Following fermentation, their abundance increased in the solid samples and decreased in the liquid samples, indicating the potential for NH_4_^+^-N release or phosphorus mineralization from organic matter. These results provide information regarding changes in nutrient and bacterial formation when applying the fermentation process after solid–liquid separation.

## Introduction

The consumption of meat has increased and is projected to grow by 48% from 2005 to 2050. This growth is driven by the rising demand for animal protein, which is a result of population growth and shifts in dietary habits due to economic growth^1–3^. As a result of increased meat consumption, the number of farms engaged in livestock farming has also increased, leading to an increase in livestock excretion worldwide^4^. These excretions release greenhouse gases such as methane (CH_4_), carbon dioxide (CO_2_), and nitrous oxide (N_2_O), which contribute to the greenhouse effect^5^.

Nutrient leaching, such as the loss of nitrates and phosphates, can be caused by an excessive supply of soil chemicals resulting from the decomposition of livestock excrements, and this has led to environmental pollution in various countries^6^. Recent studies have confirmed the presence of antibiotics, chemicals, and heavy metals, such as zinc and copper, in livestock manure, highlighting the urgent need for effective waste management systems^7,8^. These issues negatively affect ecosystems and human health^9–11^. Therefore, it is necessary to develop effective methods to reduce and recycle livestock excrements. Manure composting has proven to be an effective recycling method for reducing the production of waste^12,13^. The composting process can be classified into three main types based on the oxygen supply: aerobic composting, anaerobic digestion, and aerobic-anaerobic coupling composting^14^. Anaerobic fermentation produces methane that can be used as biogas, whereas aerobic fermentation can promote the utilization of organic matter (OM) by microorganisms^15^. Anaerobic digestion of animal waste allows simultaneous energy recovery in biogas production, and the liquid end-product can provide bioavailable nutrients such as nitrogen (N), phosphorus (P), and potassium (K) that can be used as fertilizers for plants^16,17^. Aerobic fermentation is effective in livestock manure compositing because of reduced time and space requirements^18^. Researchers are attempting to enhance aerobic fermentation techniques to address environmental pollution caused by livestock excrement waste. In addition, adding compost to soil can have a positive impact on its quality^19^.

Microorganisms play a crucial role in sustainable agriculture due to their positive effects on plant growth^20,21^. Fungal growth on plant roots increases the formation of *Chitinophagaceae* and *Flavobacteriaceae*, which suppress diseases in the plant roots^10^. Furthermore, microorganisms that could promote plant growth, i.e., plant growth-promoting bacteria (PGPB), have been investigated^22,23^. PGPB possess 1-aminocyclopropane-1-carboxylate deaminase, which is the main mechanism for reducing plant ethylene levels and promoting plant growth^24^. In a previous study, the application of *Pseudomonas* BA-152, *Bacillus* OSU-3, and *Bacillus* M-3 increased the production, growth, and nutrient content of strawberries^25^. In the intercropping of fennel and common beans using PGPB, crop production increased by 24%^26^. Additionally, diverse soil microorganisms such as *Pseudomonas*, *Bacillus*, and *Rhizobium* contribute to N mineralization and phosphate solubilization^27,28^. The hydrolytic enzymes produced by microorganisms also play a role in reducing heavy metals such as nickel, copper, lead, cobalt, zinc, and cadmium in the soil^29^. In agricultural settings, microbial extracellular polymeric substances (EPS) are utilized to provide protection against environmental stresses such as salinity and drought. They enhance soil particle aggregation, maintain moisture, and promote trapping of nutrients^30^. Improved pore connectivity can facilitate the smooth movement of water, air, and nutrients in the soil^31^. These results emphasize that microorganisms play a positive role in improving plant development. Thus, the application of microorganisms in agriculture may be useful for reducing the use of chemical fertilizers^32,33^.

Microorganisms have diverse effects on the quality and efficacy of livestock manure compost^34^. Microbial activity was enhanced by humic substances derived from agricultural waste in the binding of CO_2_^35^. The addition of exogenous humic substances can be considered a soil conditioner that enhances soil structural stability^36^. Microbial enzymes for OM decomposition improve the utilization of chemical properties such as N, P, and K to provide plant nutrients for agricultural activity^37–40^. Dominant bacteria were observed in the composting of livestock manure through selection, competition, and cooperation of microorganisms^41,42^. Industrial fertilizer production processes can involve the initial separation of liquid and slurry from livestock excretions before fermentation for the rapid production of organic fertilizer. In this study, our objective was to compare the diversity of bacterial communities in solid and liquid manures before and after fermentation, as well as in mixed livestock manure prior to solid–liquid separation. We also related bacterial abundance within the samples to their chemical properties. Our results provide useful information for the production process of organic fertilizers.

## Results

### Chemical properties of solid and liquid samples

The chemical properties of the solid and liquid samples were determined before and after fermentation during livestock composting (Table 1). Many chemical properties, except for pH, NH_4_^+^-N, Fe, Mo, and OM, were increased in FSM compared to NSM. The EC, N, NH_4_^+^-N, and P were decreased in FLM compared to NLM, while NO_3_^-^-N was increased. Many chemical properties showed no significant differences between FLM and NLM, unlike the solid samples before and after fermentation. Significant correlations were identified between the chemical properties of the two samples (Fig. S1). For instance, the pH was negatively correlated with the EC (*r* =  − 0.738), N (*r* =  − 0.636), and K (*r* =  − 0.725). In contrast, the EC was positively correlated with N (*r* = 0.974), K (*r* = 0.839), Na (*r* = 0.757), and Cl (*r* = 0.634). This result suggests that high concentrations of K, Na, and Cl led to a decrease in the EC of the liquid samples. Additionally, a high pH was found to be related to the EC, N, and K levels in the solid samples. Among the solid samples, OM decreased in the FSM owing to fermentation. However, many chemical properties, except for Fe and Mo, increased in the FSM, suggesting that OM underwent biochemical transformation and decomposition. Furthermore, the TN content significantly increased in the FSM system owing to the continuous N supply. The concentration of NO_3_^−^-N increased in the FSM and FLM owing to nitrification, where NH_4_^+^-N was converted to NO_3_ during fermentation. However, TN and NH_4_^+^-N significantly decreased in the FLM, unlike in the FSM. No difference in chemical properties was observed between the NLM and FLM. We confirmed that a sufficient quantity of OM is important for the continuous supply of N and nitrification of organic fertilizers during fermentation.

**Table 1:** Chemical properties of solid and liquid samples before and after fermentation.

Chemical composition	NSM	FSM	NLM	FLM
pH	9 ± 0.4a	9.4 ± 0.2a	7.8 ± 0.2b	7.5 ± 0.2b
EC (dS/m)	1.6 ± 0.1d	2.7 ± 0.2c	18.6 ± 0.2a	9.3 ± 0.2b
N (mg/kg)	600.2 ± 21.4d	1050 ± 217c	2380.1 ± 89.3a	1450.9 ± 43.8b
NH_4_^+^-N (mg/kg)	1,239.4 ± 18.2b	1287.9 ± 23.2b	1666.3 ± 41.4a	204.8 ± 28.7c
NO_3_^−^-N (mg/kg)	397.5 ± 26c	482.9 ± 24.2b	15 ± 4.2d	629.1 ± 36.3a
P (mg/kg)	270.4 ± 18.4b	520.1 ± 48a	266.8 ± 24.2b	31 ± 4.9c
K (mg/kg)	240.3 ± 22.5c	750.3 ± 48.2b	1295.4 ± 38.1a	1228.2 ± 42.7a
Na (mg/kg)	170.4 ± 18.8c	290.3 ± 18.6b	349.9 ± 23.1a	314.1 ± 36.7ab
Cl (mg/kg)	330.4 ± 23.3b	660.2 ± 42.3a	712.2 ± 37.5a	694.1 ± 48.2a
Ca (mg/kg)	6483.5 ± 765b	11,939.5 ± 1892a	542.4 ± 48.8c	102.6 ± 32.5c
Mg (mg/kg)	1364.8 ± 130b	3726 ± 819a	165.3 ± 18.2c	45.2 ± 21.1c
Al (mg/kg)	87.2 ± 18.7b	321.5 ± 18a	0 ± 0c	0 ± 0c
Fe (mg/kg)	2574.9 ± 328a	1810.3 ± 204b	52.3 ± 18.9c	26 ± 11.3c
Mo (mg/kg)	6 ± 1.2a	6.6 ± 0.8a	4.1 ± 0.6b	4.2 ± 0.9b
Mn (mg/kg)	93.1 ± 4.4b	175.3 ± 18.8a	5.8 ± 0.8c	0 ± 0c
SO_4_ (mg/kg)	3982.5 ± 827b	6714.8 ± 1081a	504.1 ± 58c	239.3 ± 27.5c
B (mg/kg)	14.1 ± 2.9b	20.3 ± 2.9a	6.9 ± 1.9c	6.3 ± 1.6c
OM (%)	36.3 ± 4.2a	23.2 ± 3.1b	1 ± 0.3c	0.3 ± 0.2c

The samples included unprocessed manure before solid–liquid separation (UM), non-fermented solid manure (NSM), fermented solid manure (FSM), non-fermented liquid manure (NLM), and fermented liquid manure (FLM). The values are presented as the mean ± standard deviation (n = 3). Lowercase letters represent significant differences (*p* < 0.05) between groups as determined using Duncan’s test.

### Soil microbial composition

**Figure 1:**
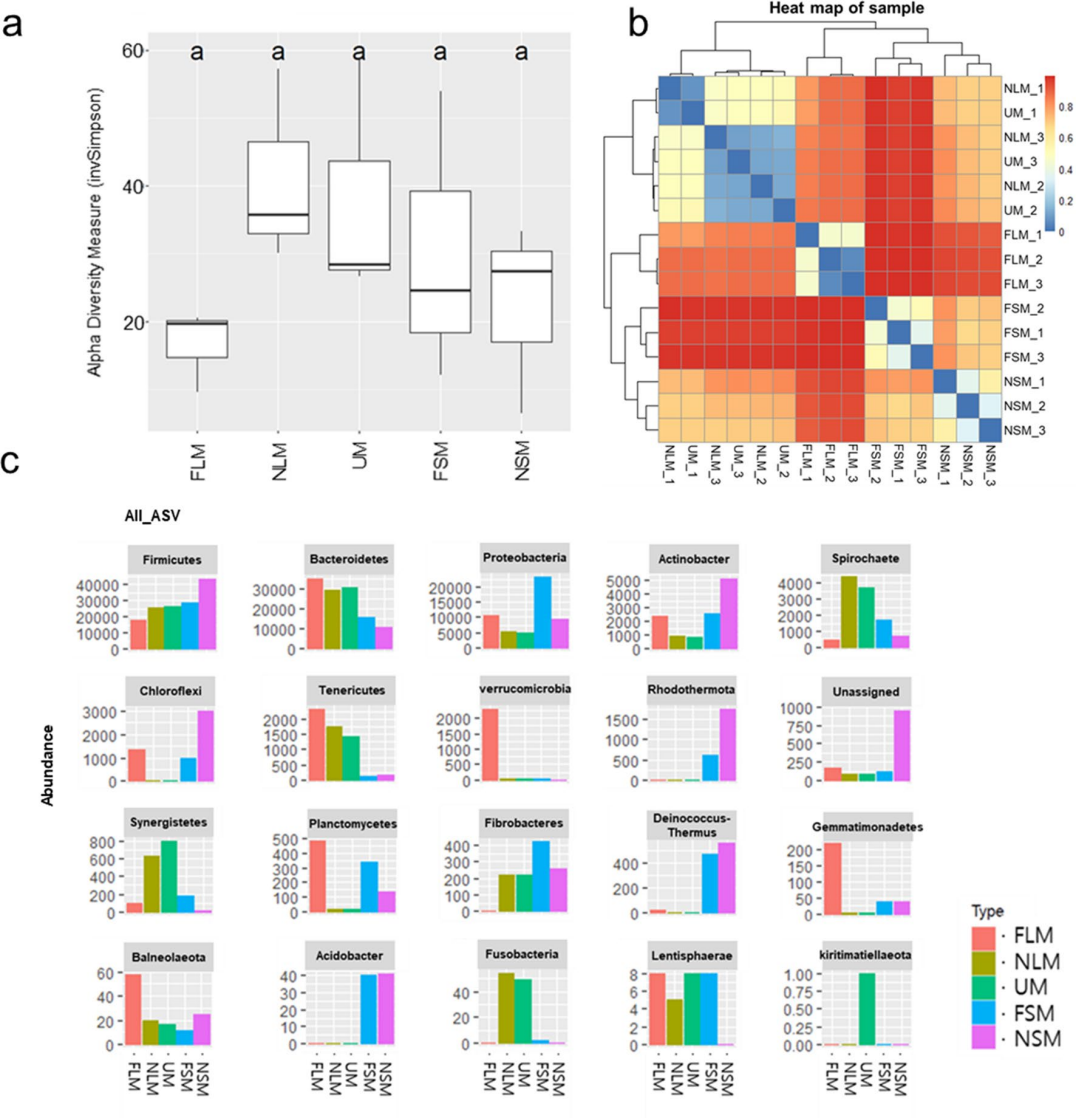
Bacterial communities in the solid and liquid samples before and after fermentation. The samples included unprocessed manure before solid–liquid separation (UM), non-fermented solid manure (NSM), fermented solid manure (FSM), non-fermented liquid manure (NLM), and fermented liquid manure (FLM). (**a**) Alpha diversity was measured using the inverse Simpson method. The letters represent significant differences (*p* < 0.05) determined by Tukey’s HSD test. The y-axis indicates the observed alpha diversities as indicated by the inverse Simpson index. (**b**) Hierarchical clustering of the beta diversity distance matrix using Bray–Curtis dissimilarity. The color represents the range of the beta diversity, which ranges from 0 (dark blue color) to 1 (dark red color). A value of 0 indicates identical communities across the samples, while a value of 1 indicates different communities among the samples. (**c**) Distribution of bacterial phyla. The bar colors represent the various samples. The y-axis indicates the number of ASVs detected in the bacterial phyla.

The diversity of microbial communities was observed in the solid and liquid samples before and after fermentation, as well as in the unprocessed manure before solid–liquid separation (UM), which is a livestock excretion used to develop solid and liquid fertilizers (Fig. 1). Although the alpha diversity, represented by the abundance of taxa in each sample, was lower in the FLM than in the NLM, there were no significant differences among the different samples (Fig. 1a). The alpha diversities of the ASVs were similar between the NSM and FSM. The beta diversity of the microbial community was estimated across the ASVs of the samples to examine the microbial relationships among the samples (Fig. 1b). The UM was highly similar to the NLM, in contrast to the solid samples (NSM and FSM), indicating that the bacteria from the raw materials were primarily present in liquid manure. The dissimilarity of liquid samples between the NLM and FLM was higher than that between the NSM and FSM. We hypothesized that fermentation leads to a reduction in the diversity of the microbial community in the FLM. *Firmicutes*, *Bacteroidetes*, and *Proteobacteria*, which had a relatively high number of ASVs, were the dominant phyla in each sample (Fig. 1c). *Acidobacteria*, *Deinococcus*-*Thermus*, and *Rhodothermaeota* were overrepresented in the solid samples (NSM and FSM) compared to the liquid samples (NLM and FLM) and UM. In contrast, *Tenericutes* were overrepresented in the liquid samples (NLM and FLM) and UM compared with the solid samples (NSM and FSM). The number of *Proteobacteria* commonly increased in the FLM and FSM samples. The diversity of several bacterial phyla was altered in solid and liquid samples during fermentation. For example, the relative abundance of *Rhodothermaeota* was lower in the FSM than in the NSM. Furthermore, the abundances of *Balneolaeota*, *Verrucomicrobia*, and *Gemmatimonadetes* increased in the FLM, whereas *Planctomycetes* were overrepresented in both the FLM and FSM compared to non-fermented samples (NLM and NSM). During fermentation, the numbers of *Spirochaetes*, *Synergistetes*, and *Fusobacteria* decreased in the FLM but increased in the FSM. *Chloroflexi* were overrepresented in the FLM and NSM samples, indicating the distinct formation of the microbial community in the process of livestock manure composting between the solid and liquid samples.

### Differentially abundant bacterial species

**Figure 2:**
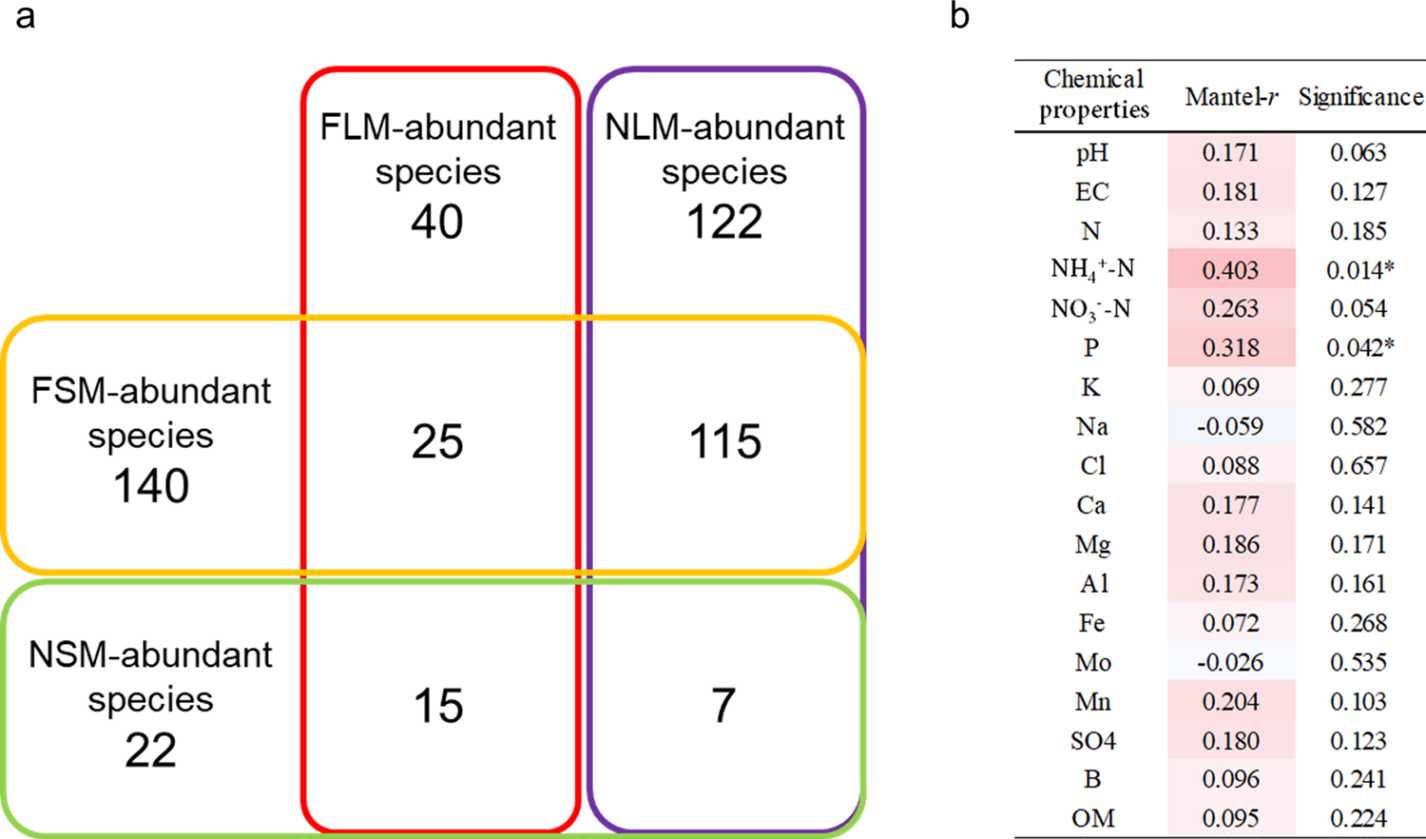
Differentially abundant bacterial species between the non-fermented and fermented samples. The samples included non-fermented solid manure (NSM), fermented solid manure (FSM), non-fermented liquid manure (NLM), and fermented liquid manure (FLM). (**a**) The number of bacterial species detected in the fermented samples is based on their relative abundance. (**b**) Mantel test between chemical properties and bacterial composition. The box color represents the different levels of correlation determined by the Mantel test (*p* < 0.05, *p* < 0.01, and *p* < 0.001).

To assess changes in the microbial community during fermentation, we determined the relative abundance of bacteria in both the liquid and solid samples (Fig. 2). Among the total number, during fermentation, 140 bacterial species (86%) increased in the solid samples with higher OM content; however, 122 bacterial species (75%) decreased in the liquid samples with lower OM content (Fig. 2a). In particular, 115 bacterial species (71%) showed distinct differences in abundance between the solid (FSM vs. NSM) and liquid (FLM vs. NLM) groups. During fermentation, the populations of many bacterial species increased in the solid samples but decreased in the liquid samples. The bacteria, which were differentially distributed, exhibited a clear distinction in their formation between the solid and liquid samples. For example, 115 bacterial species were abundant in the FSM, but not in the FLM. Fifteen bacterial species were abundant in the FLM but not in the FSM. Thirty-two bacterial species increased or decreased in both the FSM and FLM. We found significant correlations (*p* < 0.05) between the differentially abundant bacteria and NH_4_^+^-N (r = 0.403) and P (r = 0.318; Fig. 2b). Although the correlation with NO_3_^−^-N was not statistically significant (*p* < 0.05), it was marginally significant (*p* = 0.054). Thus, we hypothesized that differentially abundant bacteria are associated with NH_4_^+^-N release, nitrification, and mineralization of OM.

**Figure 3:**
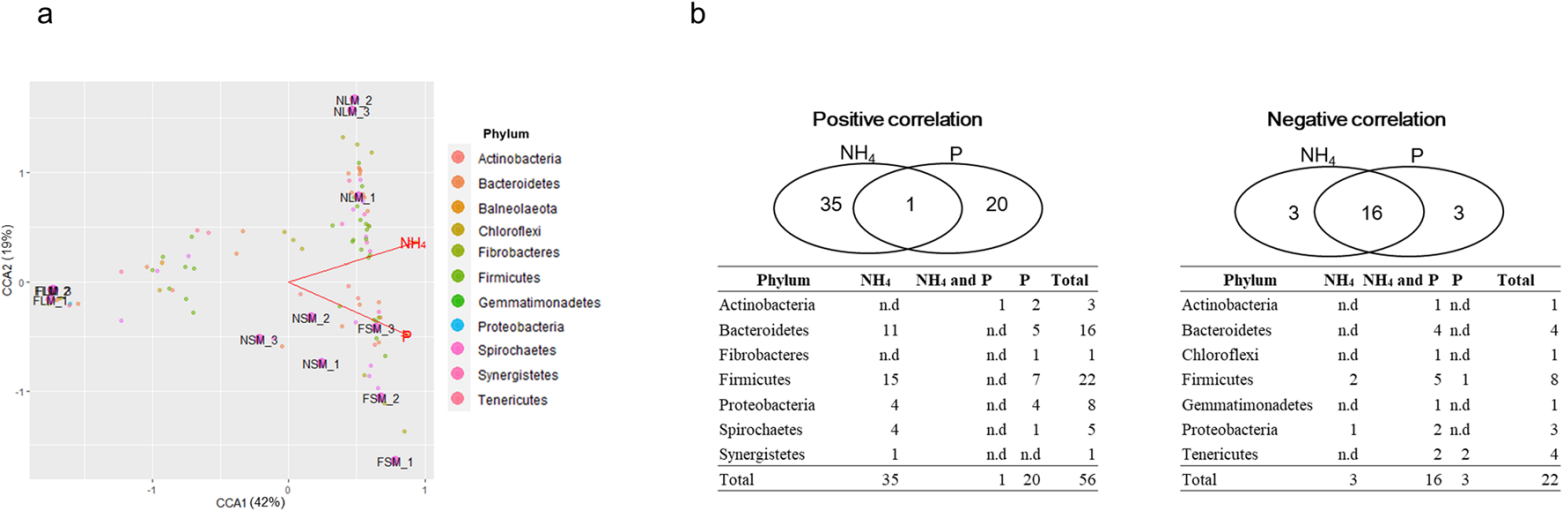
Bacterial species significantly correlated with NH_4_^+^-N and/or P. (**a**) CCA plot of samples (NSM, non-fermented solid manure; FSM, fermented solid manure; NLM, non-fermented liquid manure; FLM, fermented liquid manure) showing the relationship between differentially abundant bacterial species and two chemical properties (NH_4_^+^-N and P). (**b**) The distribution of bacterial species significantly correlated with NH_4_^+^-N and/or P (*n.d.* not detected).

In the CCA plot, the explained variation was 42% for CCA1 and 19% for CCA2. NH_4_^+^-N was an important factor in the formation of the microbial community for the NLM group, while P was the main influencing factor for the NSM and FSM groups (Fig. 3a). We observed a significant correlation of 78 bacterial species of all phyla and NH_4_^+^-N or P. Among those, 56 bacterial species exhibited a positive correlation and 22 species showed a negative correlation (Figs. 3b and S2). *Firmicutes* (22 species) and *Bacteroidetes* (16 species) were relatively abundant and showed a positive correlation with NH_4_^+^-N and P. Furthermore, 35 species in five phyla (*Bacteroidetes*, *Firmicutes*, *Proteobacteria*, *Spirochaetes*, and *Synergistetes*) were positively correlated with NH_4_^+^-N, and 20 species in six phyla (*Actinobacteria*, *Bacteroidetes*, *Firmicutes*, *Proteobacteria*, and *Spirochaetes*) were positively correlated with P. *Corynebacterium xerosis* commonly showed a positive correlation with NH_4_^+^-N and P (Fig. S2). However, eight species of *Firmicutes* were negatively correlated with NH_4_^+^-N and P. Sixteen bacterial species were commonly negatively correlated with NH_4_^+^-N and P (Fig. S2). These findings suggest that the specific composition of the bacterial community, characterized by a decrease in the quantity of several bacterial species in the liquid samples, was a result of the reduction in NH_4_^+^-N and P levels during fermentation.

### Microbial community changes during fermentation

**Figure 4:**
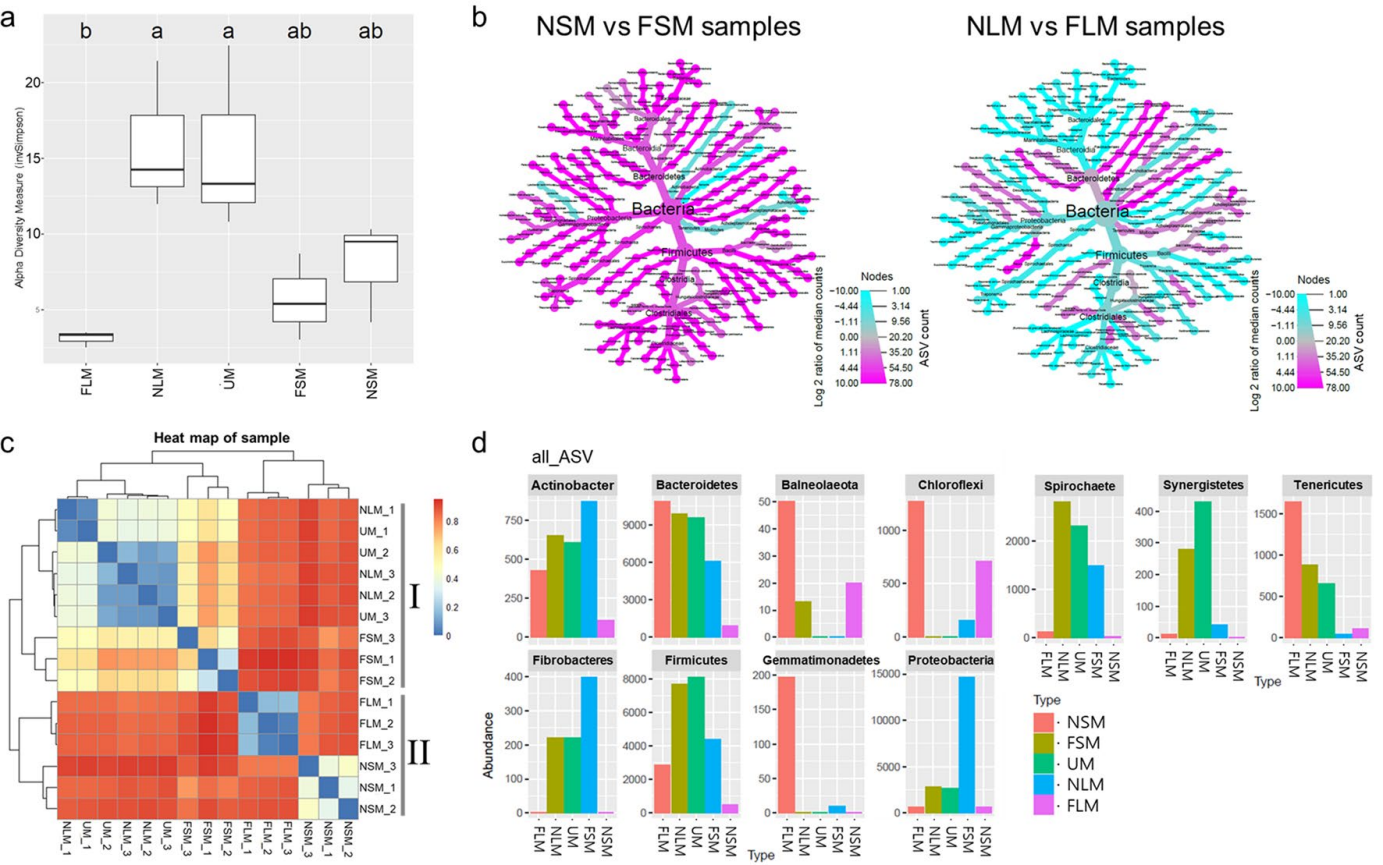
Microbial community of bacterial species significantly correlated with NH_4_^+^-N and/or P for unprocessed manure before solid–liquid separation (UM), non-fermented solid manure (NSM), fermented solid manure (FSM), non-fermented liquid manure (NLM), and fermented liquid manure (FLM). (**a**) Alpha diversity of 78 bacterial species based on the inverse Simpson method. The letters represent significant differences (*p* < 0.05) determined by Tukey's HSD test. (**b**) Relative abundances of bacteria significantly correlated in the FSM and FLM samples compared to the non-fermented samples. The color indicates the log twofold change of ASVs with a p-adjust value of less than 0.05. (**c**) Hierarchical clustering of the beta diversity distance matrix using Bray–Curtis dissimilarity. The color represents the range of the beta diversity, which ranges from 0 (dark blue color) to 1 (dark red color). A value of 0 indicates identical communities across the samples, while a value of 1 indicates different communities among the samples. Groups were determined in the hierarchical clustering tree by the k-means algorithm with a value of 2: Group I for the UM, NLM, and FSM, and Group II for the NSM and FLM. (**d**) Distribution of bacterial phyla based on 78 bacterial species. The bar colors represent the various samples. The y-axis indicates the number of ASVs detected in the bacterial phyla.

We conducted a more in-depth analysis of the specific composition of bacterial species in relation to fermentation in both liquid and solid samples (Fig. 4). Alpha diversity was assessed for the 78 bacterial species that showed a significant correlation with NH_4_^+^-N and/or P (Fig. 4a). The alpha diversity was similar between the NLM and UM, and the solid samples had lower alpha diversity than the UM. However, no significant differences were observed among the different samples although the FLM had lower alpha diversity. The abundances of the 78 bacterial species were significantly different between the solid and liquid samples after fermentation (Fig. 4b).

After fermentation, the abundance of many bacterial species increased in the solid samples and decreased in the liquid samples. We hypothesized that the diversity of microbial communities may be divided into two groups based on the increase or decrease of bacterial diversity during fermentation. In the hierarchical clustering with 2 k-means, the ASVs of the bacterial species were divided into two groups: Group I, which included the UM, NLM, and FSM samples; and Group II, which included the NSM and FLM samples (Fig. 4c). In the 78 bacterial species, the beta diversity between the UM and FSM was lower than that between the UM and NSM, while the beta diversity between the UM and FLM was higher than that between the UM and NLM. Many bacterial phyla showed a significant correlation with NH_4_^+^-N and/or P and exhibited distinct compositions in the solid and liquid samples during fermentation (Fig. 4d). For example, *Actinobacteria*, *Spirochaetes*, *Synergistetes*, *Fibrobacteres*, *Firmicutes*, and *Proteobacteria* were relatively abundant in the FSM but not in the FLM. Although *Balneolaeota*, *Chloroflexi*, and *Tenericutes* were relatively abundant in the FLM, they were not as prevalent in the FSM. The abundance of *Bacteroidetes* and *Gemmatimonadetes* increased in the FSM and FLM. These results suggest that a distinct development of the microbial community occurred in the solid and liquid samples owing to a deficiency in NH_4_^+^-N and/or P.

## Discussion

**Figure 5:**
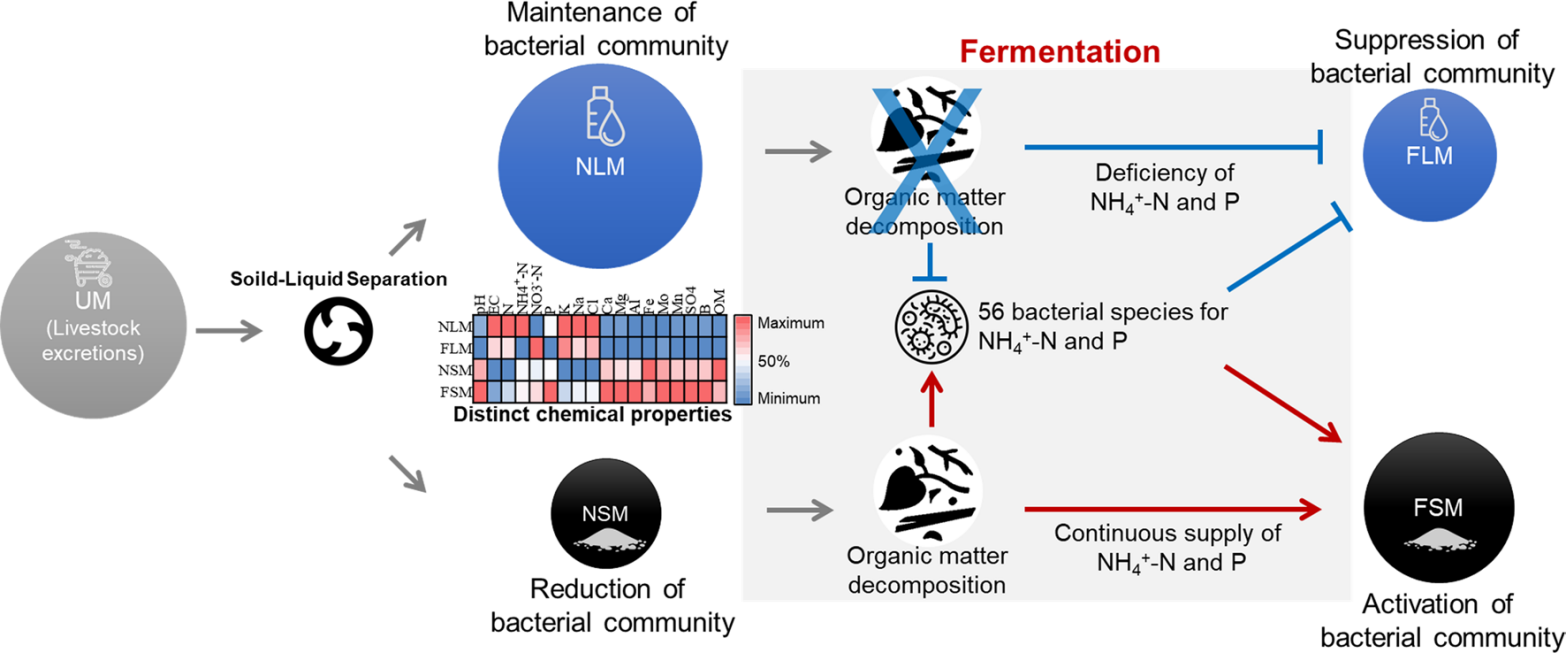
Distinct development of microbial communities between solid and liquid samples during fermentation. The samples included unprocessed manure before solid–liquid separation (UM), non-fermented solid manure (NSM), fermented solid manure (FSM), non-fermented liquid manure (NLM), and fermented liquid manure (FLM). The size of the circles represents the level of bacterial abundance. The color of the circles represents the different samples.

Distinct chemical properties were identified among the various samples during the manure composting process (Fig. 5). We found that the pH of the liquid samples was lower than that of the solid samples. The low pH may have been due to the effluent of organic acids from the solid into the liquid samples during the initial stage of composting. A decrease in pH due to microbial metabolism has been observed in different composting samples containing crop straw^43^. The pH is a major factor in the formation of bacterial communities owing to changes in nutrient availability. Here, the solid and liquid manures exhibited different nutrient accumulations through solid–liquid separation prior to fermentation. In a previous study, soil samples collected from Polish arable lands had higher NH_4_^+^-N and lower NO_3_^−^-N concentrations because of a decreasing soil pH^44^. The NO_3_ uptake system in plants is activated by increased leaching of nitrate (NO_3_^−^) caused by higher soil pH^45^. Thus, the higher pH of the NSM group may have affected the increase in NO_3_^−^-N. Furthermore, the liquid sample showed an increase in several organic manure components, including N, NH_4_^+^-N, K, and Na, whereas the solid sample showed an increase in Ca, Mg, Al, Fe, Mo, Mn, SO_4_, B, and OM. In particular, the contents of Ca, Mg, Al, Mo, Mn, SO_4_, and B increased in the solid sample during fermentation, suggesting the biochemical transformation of OM, which decreased after fermentation. Solid manure contains higher amounts of OM than liquid manure^46^. The leachate generated from livestock contains large amounts of nutrients, such as ammonia; thus, it can be used as organic fertilizer for plants and microalgae after a suitable manufacturing process because of the enhancement of soil fertility^47,48^. The chemical properties, except for total N, NH_4_^+^-N, and NO_3_^−^-N, did not significantly differ between the liquid samples before and after fermentation. N transformation of organic carbon was observed during the process of nitrification from NH_4_^+^-N to NO_3_^−^-N and denitrification from NO_3_^−^-N to N_2_^49,50^.

In this study, we found a decrease in NH_4_^+^-N and an increase in NO_3_^−^-N in the liquid sample after fermentation (FLM), indicating the nitrification of N compounds. The concentration of NO_3_^−^-N increased in the solid sample after fermentation (FSM); however, there was no decrease in the concentration of NH_4_^+^-N. OM is the main source of available N for plants through the decomposition of complex organic molecules^51^. OM contributes to the release of NH_4_^+^-N in the mineralization of manure to inorganic N (ammonia and ammonium) through microbial activity^44^. OM was significantly enriched in the NSM compared to the NLM, and OM was significantly decreased in the FSM compared to the NSM. However, the FLM did not show a significant decrease in OM compared to the NLM. This result suggests that the amount of OM was sufficient to release NH_4_^+^-N into the solid samples during fermentation.

**Figure 6:**
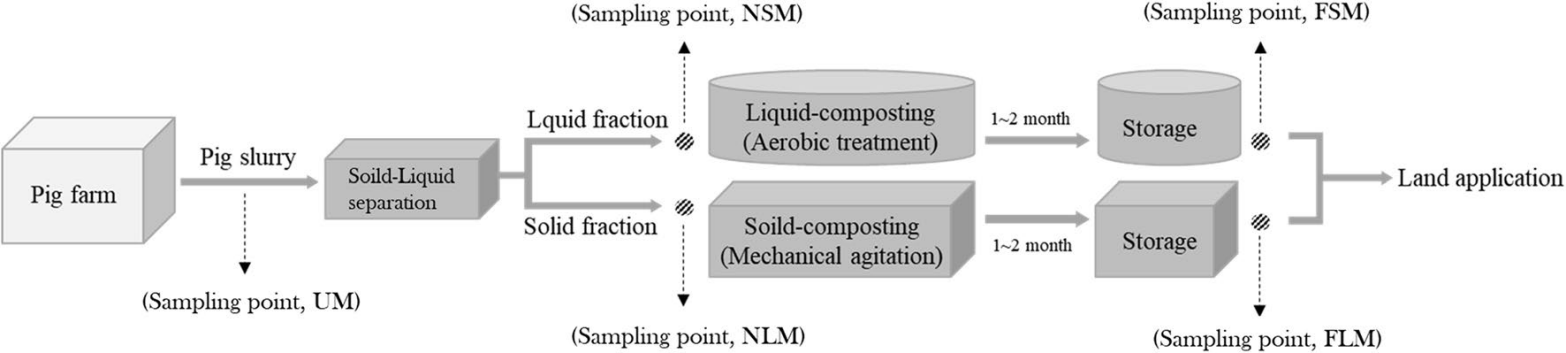
Schematic layout of the manure management process at the pig farm in this study. The samples included unprocessed manure before solid–liquid separation (UM), non-fermented solid manure (NSM), fermented solid manure (FSM), non-fermented liquid manure (NLM), and fermented liquid manure (FLM).

Microbial mineralization of N from OM is necessary for crop production in soil, and biological N-fixation by microorganisms can improve the N content of soil by 30–50 kg/ha/year^52^. The 56 bacterial species in six phyla (*Actinobacteria*, *Bacteroidetes*, *Firmicutes*, *Proteobacteria*, *Spirochaetes*, and *Synergistetes*) had a significant positive correlation with NH_4_^+^-N and/or P. Wang et al.^53^ reported that an increase in NH_4_^+^ ions led to soil acidification, resulting in a decrease in soil pH. Thus, the change in pH caused by NH_4_^+^ may affect the microbial community. However, we did not find any statistical significance (*p* < 0.05) in the relationship between the pH and the development of the microbial community, despite the relatively low p-value. It is necessary to consider the cross effects of chemical properties on the microbial community. After fermentation, microbial abundance increased in the solid samples and decreased in the liquid samples, suggesting the functional possibility of NH_4_^+^-N release or P mineralization from OM (Fig. 6 and Fig. S2). The contents of NH_4_^+^-N, TN, and P, as dominant predictors, contribute to a diverse composition of the bacterial community in soil^54,55^. *Actinobacteria* play important roles in plant residue decomposition and soil nutrient cycling, and the interaction between *Actinobacteria* and other microbes in eutrophic soils has a neutral effect^56–58^. Furthermore, the available phosphate in rock P increases as a result of P mineralization by *Actinobacteria* belonging to the *Streptomyces* and *Nocardiopsis* genera^59^. The *Corynebacterium* and *Pseudomonas* genera improved phosphate solubilization in sediment samples from continental slopes^60^. *Bacteroidetes*, *Firmicutes*, and *Proteobacteria* promoted N loss from soil in a ten-year field experiment^61^. Wang et al.^55^ reported that the most abundant bacteria in N/P-input and P-input soils were *Proteobacteria* and *Actinobacteria* and *Acidobacteria*, respectively, compared to no fertilizer-treated soils. P-solubilizing bacteria (PSB) improve the availability of P in plants. *Acinetobacter pittii* and *Pseudomonas extremaustralis* are PSB species^62^.

Furthermore, there were distinct differences in the abundances of several bacteria between the solid and liquid samples after fermentation (Fig. 4). In particular, the abundance of *Actinobacteria*, *Fibrobacteres*, *Firmicutes*, *Spirochaetes*, and *Proteobacteria* increased in the fermented solid samples, but decreased in the fermented liquid sample. This suggests that these bacterial phyla may have contributed to organic decomposition. *Actinobacteria* are filamentous bacteria associated with the decomposition of organic substances^63^. *Fibrobacteres*, a group of cellulose-degrading bacteria, has been found in anaerobic reactors and rice paddy soil and is considered to be involved in the decomposition of soil OM^64–66^. *Firmicutes*, which are involved in the degradation of nitrogenous organic compounds, are the dominant microorganisms in the composting process at both the early and mature stages^67^. *Spirochaetes*, *Firmicutes*, and *Actinobacteria* have been detected during the decomposition of rice straw compost^68^. During microbial mineralization, *Proteobacteria* exhibit a positive correlation between the relative bacterial abundance and the soil organic carbon content, which is controlled by microorganisms^69,70^. *Proteobacteria* and *Firmicutes* play roles in the decomposition cellulose, lignocellulose, and hemicelluloses, and their increased relative abundances promote the decomposition of soil OM^71,72^. The OM content decreased in the FSM group. Similarly, the increased activity of microorganisms in decomposing OM leads to a decrease in the soil OM content^72^. We identified several phyla possibly associated with NH_4_^+^-N release, P mineralization, and solubilization of OM in the ASV analysis. However, accurate studies regarding the functional relationship of bacteria and chemical properties at the species level are absent. Thus, more studies are needed to demonstrate the reliable functionality of the bacterial species in this study. The application of solid–liquid separation offers numerous advantages in the treatment of livestock manure. For example, the solid fraction was able to provide soil nutrients in cultivated land that was deficient in nutrients^73^. For efficient nutrient transport to arable farms, it is necessary to reduce the volume of manure, which consists of a mixture of solid and liquid matter, due to the high cost of transportation^74^. Thus, solid–liquid separation can reduce transportation costs because the solid fraction has a much smaller volume than the liquid fraction. The liquid fraction after the removal of solid materials can be used for biogas production and as wash water^73,75^. Furthermore, the liquid fraction has the advantage of being both inexpensive and easy to treat^76^. Thus, production of solid and liquid manure needs to consider the development of distinct bacterial communities due to changes in chemical properties during the fermentation process after solid–liquid separation.

In this study, we evaluated differences in the chemical properties and bacterial communities of solid and liquid samples before and after fermentation. Before fermentation, several chemical properties, such as the EC, total N, NH_4_^+^-N, NO_3_^−^-N, K, Na, and Cl, as well as the relative abundance of bacteria were higher in the liquid than the solid samples. After fermentation, the liquid samples showed a decrease in many chemical properties and relative bacterial abundance, which could be attributed to the low OM content. In contrast, the contents of chemicals in the solid samples increased. Furthermore, we found that 56 bacterial species may have been responsible for changes in NH_4_^+^-N and P contents. Finally, the relatively most abundant bacteria in the solid samples after fermentation were related to the decomposition of OM. These results help understand changes in bacterial abundance and composition and chemical properties of livestock manure during the fertilizer production process after solid–liquid separation.

## Methods

### Sampling in the process of livestock manure composting

Samples were collected from a pig farm (37°13′14.3" N, 127°24′27.8" E) located in Icheon-si, Gyeonggi-do, Republic of Korea specializing in producing piglets on a scale of 5000 heads. The pig farm uses an aerobic treatment process to compost liquid and solid manure. An inclined screw press (HK-3000BK, HKED, Korea) was used to separate solid and liquid manure. After 30 min of solid–liquid separation, samples of non-fermented solid manure (NSM) and non-fermented liquid manure (NLM) were collected on the same day in three experimental replicates (Fig. 6). The fermentation process lasted for two months and was conducted in an indoor composting room at an average temperature of 20 °C. One hundred fifty tons of liquid manure was fermented in a liquid manure tank by an air ring blower at 0.03–0.045 m^3^-air/min·m^3^, and 14 t of solid manure was fermented in a solid manure-composting ground by mechanical agitation once per day. Fermented solid manure (FSM) and fermented liquid manure (FLM) were collected on the same day in three experimental replicates during the final storage stage for land application. The maturity of the compost was evaluated using the Solvita method^77^. We confirmed a maturity index of 8 in FSM and FLM after two months of fermentation. The collected manures were stored in a deep freezer (NF-400SF, TAESHINBIO, Korea) at -80 °C for further experiments. The sampling method adhered to the quality inspection method and sample collection criteria for fertilizers in South Korea.

### Chemical components of manure

The chemical components (pH, electric conductivity [EC], total nitrogen [TN], ammonium nitrogen [NH_4_^+^-N], nitrate nitrogen [NO_3_^−^-N], and OM) of the solid and liquid in manure before or after fermentation were analyzed using the method described in a previous study by Lee et al.^19^. The P, K, sodium (Na), chlorine (Cl), sodium chloride (NaCl), calcium (Ca), magnesium (Mg), aluminum (Al), iron (Fe), molybdenum (Mo), manganese (Mn), sulfate (SO_4_^2−^), and boron (B) contents were analyzed using previously utilized methods^78^.

### Microbiome analysis

To survey microbial diversity in the production process of livestock manure, we performed 16S rRNA sequencing analysis in solid and liquid manures after separation of livestock excretion, following the method used by Lee et al.^19^. In summary, soil microbial DNA was extracted using the DNeasy PowerSoil Kit (Qiagen, Hilden, Germany) immediately after sampling. Subsequently, a polymerase chain reaction (PCR) targeting the soil microbiome's 16S rRNA gene regions V3-V4 was performed using Herculase II Fusion DNA Polymerase (Agilent Technologies, Santa Clara, CA, USA). To amplify the V3-V4 16S rRNA regions, the primer pairs used for the first amplification were as follows: V3-F: 5′-TCGTCGGCAGCGTCAGATGTGTATAAGAGACAGCCTACGGGNGGCWGCAG-3′, V4-R: 5′-GTCTCGTGGGCTCGGAGATGTGTATAAGAGACAGGACTACHVGGGTATCTAATCC-3′.

16S rRNA sequencing was performed using the Illumina NovaSeq 6000 system^79^. The adapter sequences from the raw sequence data (FASTQ file) were trimmed using the Cutadapt program with the default parameters^80^. The 16S ribosomal RNA sequences were downloaded from the National Center for Biotechnology Information (NCBI) database (https://www.ncbi.nlm.nih.gov/). The Amplicon sequence variants (ASVs) were analyzed by the QIIME and DADA2 pipelines using the default parameters^81,82^. The ASVs were normalized by the R package SRS (https://cran.r-project.org/web/packages/SRS/index.html) with the ranked subsampling method. The taxonomy of ASVs was identified using the Basic Local Alignment Search Tool (BLAST) program with the blastn algorithm and an e-value < 1e-10^83^. The taxonomic diversity and abundance of ASVs were analyzed using several R packages, including dplyr^84^, taxa^85^, ape^86^, ggrepel^87^, pyloseq^88^, and ggsignif^89^. The distribution of ASVs among samples was assessed using the R package DESeq2 with a False Discovery Rate (FDR) < 0.05^90^.

### Statistical analysis

To test significant differences in chemical properties, analysis of variance (ANOVA) was conducted using the R function “aov” (https://www.rdocumentation.org/packages/stats/versions/3.6.2/topics/aov). Duncan's test at a significance level of 0.05 was then applied on the aov output using the R package agricolae (https://cran.r-project.org/web/packages/agricolae/index.html). The alpha diversity of microorganisms within the samples was estimated based on the inverse Simpson index using the R package vegan^91^. Significant differences in alpha diversities were tested using ANOVA using the R function “aov”, followed by Tukey's honestly significant difference (HSD) test using the R function “HSD.test” (https://www.rdocumentation.org/packages/ agricolae/versions/1.3–7/topics/HSD.test) with the default parameters. The beta diversity among samples was analyzed using the R package “vegan”^91^ with Bray–Curtis dissimilarity. The beta diversity results were then visualized using the R package “pheatmap” (https://www.rdocumentation.org/packages/pheatmap/versions/1.0.12/topics/pheatmap). To investigate significant correlations between soil chemical constituents and bacterial communities, we performed a Mantel test using the R package “vegan”^91^ with 9,999 permutations. The significance of Pearson’s correlation coefficient was determined using n-2 degrees of freedom. We further analyzed the relationship between significant chemical properties and differentially abundant bacterial species using Canonical Correlation Analysis (CCA). CCA was performed by using the R package “CCA” (https://cran.r-project.org/web/packages/CCA/index.html).

### Supplementary Information

Supplementary Figures.

## Data Availability

Raw reads from isolates sequenced in this study are available at the NCBI Short Read Archive (SRA) under BioProject accession no. PRJNA1013211 (https://www.ncbi.nlm.nih.gov/bioproject/PRJNA1013211).
