# Supplementary material for: Analysis of microbial communities in solid and liquid pig manure during the fertilization process
Source: Sci Rep. 2024 Jan 2;14:72. doi: 10.1038/s41598-023-50649-5 (PMC10761828; doi:10.1038/s41598-023-50649-5)
Supplement: Supplementary file 1 — Supplementary Figures. [file 41598_2023_50649_MOESM1_ESM.pdf]

Analysis of microbial communities in solid and liquid pig manure during the fertilization process

Soo-Ryang Kim [1†], Junkyung Lee [2†], Myung Gyu Lee [3], Ha Guyn Sung[4], Sun-Goo Hwang[3*]

[1]Industry- Academic Cooperation Foundation, Sangji University, Wonju 26339, Republic of Korea

[2]Dept. of Applied Plant Science, Sangji University, Wonju-si 26339, Republic of Korea

[3]Dept. of Smart Life Science, Sangji University, Wonju-si 26339, Republic of Korea

[4]Animal Feeding and Environment Laboratory, Dept. of Animal Science, Sangji University, Wonju-si 26339, Republic of Korea

[†]These authors contributed equally to this work and share the first authorship.

*** Correspondence:**

Sun Goo Hwang

sghwang9@sangji.ac.kr

	pH	EC	N	NH_4^+-N	NH_3^--N	P	K	Na	Cl	Ca	Mg	Al	Fe	Mo	Mn	SO4	B
EC	-0.738																
N	-0.636	0.974															
NH_4^+-N	n.s	n.s	n.s														
NH_3^--N	n.s	-0.679	-0.644	-0.821													
P	0.840	n.s	n.s	0.688	n.s												
K	-0.725	0.839	0.879	n.s	n.s	n.s											
Na	n.s	0.757	0.854	n.s	n.s	n.s	0.935										
Cl	n.s	0.634	0.754	n.s	n.s	n.s	0.914	0.974									
Ca	0.953	-0.748	-0.611	n.s	n.s	0.872	-0.637	n.s	n.s								
Mg	0.891	-0.653	-0.485	n.s	n.s	0.886	-0.478	n.s	n.s	0.980							
Al	0.870	-0.638	-0.478	n.s	n.s	0.873	n.s	n.s	n.s	0.958	0.982						
Fe	0.877	-0.846	-0.831	n.s	n.s	0.574	-0.960	-0.813	-0.785	0.793	0.660	0.603					
Mo	0.933	-0.706	-0.593	n.s	n.s	0.689	-0.625	n.s	n.s	0.856	0.806	0.763	0.803				
Mn	0.946	-0.754	-0.622	n.s	n.s	0.872	-0.641	n.s	n.s	0.998	0.977	0.963	0.787	0.834			
SO4	0.968	-0.756	-0.624	n.s	n.s	0.863	-0.659	n.s	n.s	0.997	0.969	0.945	0.818	0.882	0.992		
B	0.974	-0.716	-0.574	n.s	n.s	0.857	-0.610	n.s	n.s	0.977	0.954	0.926	0.792	0.939	0.967	0.984	
OM	0.848	-0.832	-0.827	n.s	n.s	0.535	-0.970	-0.836	-0.817	0.755	0.616	0.553	0.998	0.778	0.748	0.782	0.756

Fig. S1 The significant Pearson's correlation coefficient among the chemical properties in both samples. The significance was determined by using the n-2 degrees of freedom with $p < 0.05$

Phylum	Species	ASV abundance				NH_4		P	
		NSM	FSM	NLM	FLM	PCC	p-value	PCC	p-value
Actinobacteria	Corynebacterium_halotolerans	4	110	83	12			0.677	0.016
	Corynebacterium_humireducens	32	128	94	11			0.648	0.023
	Corynebacterium_xerosis	0	18	61	0	0.646	0.023	0.785	0.002
Bacteroidetes	Alkaliflexus_imshenetskii	5	30	104	297			0.683	0.014
	Bacteroides_coprosuis	0	31	73	0	0.608	0.036		
	Bacteroides_gallinarum	0	22	200	0	0.683	0.014		
	Bacteroides_graminisolvens	0	52	358	0	0.584	0.046		
	Bacteroides_uniformis	0	157	168	0	0.612	0.034		
	Empedobacter_stercoris	0	10	23	9	0.582	0.047		
	Fermentimonas_caenicola	0	132	73	0	0.580	0.048		
	Geofilum_rhodophaeum	267	68	15	23			0.722	0.008
	Labilibacter_sediminis	0	27	9	0	0.608	0.036		
	Maribellus_luteus	0	25	110	33	0.627	0.029		
	Microbacter_margulisiae	814	80	28	0	0.622	0.031		
	Myroides_guanonis	90	680	235	37			0.598	0.040
	Parabacteroides_goldsteinii	0	6	15	41	0.670	0.017		
	Petrimonas_mucosa	0	10	33	4			0.704	0.011
	Phocaeicola_paurosaccharolyticus	34	70	0	40	0.645	0.024		
	Roseimarinus_sediminis	0	24	121	19			0.779	0.003
Fibrobacteres	Fibrobacter_succinogenes	0	58	132	23			0.746	0.005
Firmicutes	Acidaminococcus_fermentans	0	23	77	10	0.624	0.030		
	Anaerocolumna_cellulosilytica	34	0	4	0	0.649	0.022		
	Atopostipes_suicloacalis	0	15	62	0	0.667	0.018		
	Caloramator_australicus	135	31	62	22	0.603	0.038		
	Clostridium_moniliforme	0	4	14	10	0.611	0.035		
	Clostridium_populeti	0	15	21	0	0.660	0.020		
	Falcatimonas_natans	0	11	22	0			0.599	0.040
	Hungateiclostridium_saccincola	0	30	38	0			0.842	0.001
	Hungateiclostridium_straminisolvens	2075	117	34	0	0.598	0.040		
	Intestinimonas_butyriciproducens	0	88	41	0	0.655	0.021		
	Kineothrix_alysoides	0	50	329	63			0.770	0.003
	Lachnoclostridium_pacaense	0	96	242	5			0.770	0.003
	Lactobacillus_ultunensis	0	3	25	3347	0.625	0.030		
	Limosilactobacillus_reuteri	37	13	0	8	0.655	0.021		
	Lutispora_thermophila	0	23	10	0	0.701	0.011		
	Lysinibacillus_boronitolerans	13	4	18	38			0.632	0.028
	Ruminococcus_albus	0	129	219	72			0.792	0.002
	Ruminococcus_gnavus	0	4	52	0	0.595	0.041		
	Sedimentibacter_saalensis	20	10	4	0	0.593	0.042		
	Terrisporobacter_petrolearius	0	9	0	34	0.625	0.030		
	Thermoclostridium_caenicola	26	0	0	5			0.695	0.012
	Tissierella_praeacuta	4	208	312	0	0.594	0.042		
Proteobacteria	Acinetobacter_indicus	0	5	5	0			0.658	0.020
	Comamonas_denitrificans	0	17	4	0	0.593	0.042		
	Desulfomicrobium_aestuarii	0	20	67	0	0.629	0.028		
	Desulfovibrio_cuneatus	5	8	18	36	0.641	0.025		
	Oblitimonas_alkaliphila	0	408	9	138			0.607	0.036
	Pseudomonas_caeni	3	137	4	0			0.605	0.037
	Psychrobacter_pasteurii	20	5	0	12			0.761	0.004
	Succinivibrio_dextrinosolvens	0	21	53	103	0.622	0.031		
Spirochaetes	Treponema_brennaborense	0	142	361	40	0.711	0.009		
	Treponema_bryantii	0	28	18	0	0.633	0.027		
	Treponema_caldarium	0	40	110	0			0.680	0.015
	Treponema_parvum	0	3	21	0	0.654	0.021		
	Treponema_zuelzerae	126	153	252	76	0.728	0.007		
Synergistetes	Cloacibacillus_porcorum	6	38	126	105	0.605	0.037		

Phylum	Species	ASV abundance				NH_4		P	
		NSM	FSM	NLM	FLM	PCC	p-value	PCC	p-value
Actinobacteria	Schaalia_naturae	0	151	278	22	-0.734	0.007	-0.581	0.048
Bacteroidetes	Leadbetterella_byssophila	0	101	518	392	-0.945	0.000	-0.795	0.002
Bacteroidetes	Sphingobacterium_cibi	4	374	2141	57	-0.670	0.017	-0.695	0.012
	Taibaiella_smilacinae	29	173	455	69	-0.929	0.000	-0.750	0.005
	Rhodohalobacter_halophilus	0	54	31	0	-0.617	0.033	-0.668	0.018
Chloroflexi	Sphaerobacter_thermophilus	0	7	203	15	-0.858	0.000	-0.696	0.012
Firmicutes	Aminipila_butyrica	0	9	13	0	-0.764	0.004	-0.766	0.004
	Desulfuribacillus_alkaliarsenatis	0	9	21	0	-0.599	0.040	-0.622	0.031
	Ercella_succinigenes	0	35	43	5	-0.739	0.006		
	Erysipelothrix_larvae	0	29	38	0	-0.652	0.021	-0.749	0.005
	Parvimonas_micra	0	36	85	141	-0.787	0.002	-0.638	0.026
	Proteiniclasticum_ruminis	31	115	668	41	-0.667	0.018		
	Syntrophomonas_zehnderi	0	9	3	162	-0.653	0.021	-0.692	0.013
	Vulcanibacillus_modesticaldus	0	18	40	0			-0.627	0.029
Gemmatimonadetes	Longimicrobium_terrae	0	43	481	0	-0.925	0.000	-0.750	0.005
Proteobacteria	Lysobacter_sediminicola	0	10	32	0	-0.631	0.028	-0.771	0.003
	Paracoccus_alkenifer	0	26	30	98	-0.650	0.022	-0.648	0.023
	Thauera_terpenica	41	164	51	55	-0.876	0.000		
Tenericutes	Acholeplasma_brassicae	0	8	0	166			-0.748	0.005
	Acholeplasma_equifetale	6	0	0	4	-0.786	0.002	-0.634	0.027
	Acholeplasma_morum	6	0	8	44			-0.723	0.008
	Acholeplasma_vituli	0	4	47	0	-0.815	0.001	-0.802	0.002

Fig. S2 The significant Pearson's correlation coefficient (PCC) between bacterial ASV and two chemical properties (NH_4^+-N and P). The color of the PCC value represents different levels of correlation, with red indicating positive correlation and blue indicating negative correlation. The p-value was calculated using the n-2 degrees of freedom
